# 243. A Laboratory Intervention to Enhance Recognition of Bacteremia in Patients with *Staphylococcus aureus* Bacteriuria

**DOI:** 10.1093/ofid/ofad500.316

**Published:** 2023-11-27

**Authors:** James J Vaillant, Supavit Chesdachai, Isin Y Comba, Hussam Tabaja, Zachary A Yetmar, Omar M Abu Saleh

**Affiliations:** Mayo Clinic, Rochester, Minnesota; Mayo Clinic, Rochester, Minnesota; Division of Public Health, Infectious Diseases, and Occupational Medicine, Department of Medicine, Mayo Clinic, Rochester, Minnesota; Mayo Clinic, Rochester, Minnesota; Mayo Clinic, Rochester, Minnesota; Mayo Clinic Rochester, Rochester, Minnesota

## Abstract

**Background:**

*Staphylococcus aureus* bacteriuria (SABU) represents 0.2–4% of all positive urine cultures. While asymptomatic colonization or infection of the urinary tract is possible, hematogenous seeding from *S. aureus* bacteremia (SAB) represents a potentially life-threatening condition. The rate of concomitant SAB is estimated to be 6.9–17.2% among those with SABU, and these patients are at higher risk of in-hospital mortality, infective endocarditis, embolic events, and vertebral osteomyelitis. Detection of SABU represents an opportunity for early detection and intervention in SAB.

**Methods:**

We conducted a retrospective analysis of clinical and microbiological characteristics of adult patients with SABU as our pre-intervention baseline. We then implemented a comment with the urine culture result in the electronic medical record (EMR) advising providers about the possibility of concomitant bacteremia. We conducted an interim analysis of early post-intervention data.

Figure 1: Study Workflow

**Figure:**
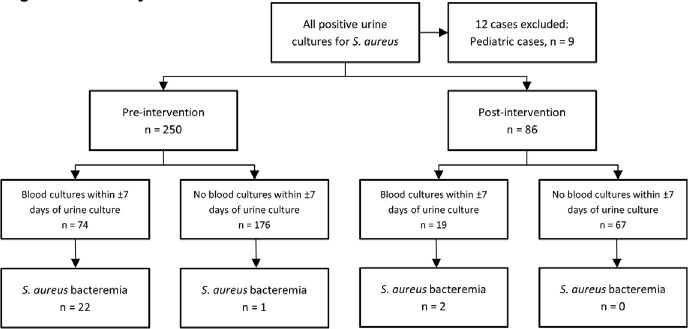

Figure 1: Study workflow depicting case recruitment and blood culture results.

Figure 2: Intervention Flag

**Figure:**
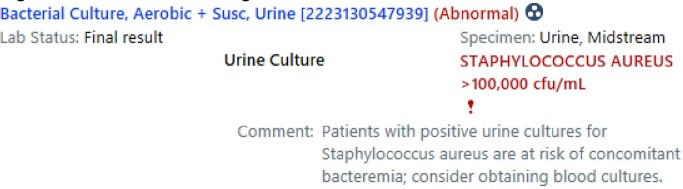

Figure 2: Example of intervention comment displayed with the urine culture result in the EMR.

**Results:**

250 adult patients developed SABU from January 1, 2021, to January 31, 2023. Of these, 87 (34.8%) had urinary tract symptoms, and 91 (36.4%) had a chronic indwelling urinary catheter. 74 (29.6%) patients had blood cultures collected within 7 days of positive urine culture. The presence of urinary symptoms or indwelling catheter was not associated with blood culture collection. Only 22 patients (8.8%) had SAB with SABU. One patient was diagnosed with SAB 20 days after detection of SABU with infective endocarditis and disseminated infection, representing a delay in diagnosis.

Following implementation of the intervention, an additional 86 patients with SABU had the result comment displayed in the EMR. Blood cultures were collected in 19 (22.1%) patients, and 2 (2.3%) were positive for *S. aureus*. There were no significant differences in rates of blood culture collection or SAB after implementation of the EMR result comment.

**Conclusion:**

Our results show a similar rate of SABU associated SAB to that previously reported in the literature. Delayed diagnosis of SAB in SABU is uncommon but does occur rarely with significant morbidity. The addition of a result comment is a simple and cost-effective intervention; however, evaluation of its efficacy is presently limited by a short follow-up duration.

**Disclosures:**

**All Authors**: No reported disclosures

